# A Global Perspective of the Genetic Basis for Carbonyl Stress Resistance

**DOI:** 10.1534/g3.111.000505

**Published:** 2011-08-01

**Authors:** Shawn Hoon, Marinella Gebbia, Michael Costanzo, Ronald W. Davis, Guri Giaever, Corey Nislow

**Affiliations:** * Department of Genetics, Stanford University, Palo Alto, California 94305; † Stanford Genome Technology Center, Palo Alto, California 94304, and; ‡ Department of Molecular Genetics, Donnelly Centre, University of Toronto, Toronto, Ontario M5S3E1, Canada

**Keywords:** glyoxal, carbonyl stress, chemogenomics, yeast deletion collection

## Abstract

The accumulation of protein adducts caused by carbonyl stress (CS) is a hallmark of cellular aging and other diseases, yet the detailed cellular effects of this universal phenomena are poorly understood. An understanding of the global effects of CS will provide insight into disease mechanisms and can guide the development of therapeutics and lifestyle changes to ameliorate their effects. To identify cellular functions important for the response to carbonyl stress, multiple genome-wide genetic screens were performed using two known inducers of CS. We found that different cellular functions were required for resistance to stress induced by methylglyoxal (MG) and glyoxal (GLY). Specifically, we demonstrate the importance of macromolecule catabolism processes for resistance to MG, confirming and extending known mechanisms of MG toxicity, including modification of DNA, RNA, and proteins. Combining our results with related studies that examined the effects of ROS allowed a comprehensive view of the diverse range of cellular functions affected by both oxidative and carbonyl stress. To understand how these diverse cellular functions interact, we performed a quantitative epistasis analysis by creating multimutant strains from those individual genes required for glyoxal resistance. This analysis allowed us to define novel glyoxal-dependent genetic interactions. In summary, using multiple genome-wide approaches provides an effective approach to dissect the poorly understood effects of glyoxal *in vivo*. These data, observations, and comprehensive dataset provide 1) a comprehensive view of carbonyl stress, 2) a resource for future studies in other cell types, and 3) a demonstration of how inexpensive cell-based assays can identify complex gene-environment toxicities.

A hallmark of aging and its attendant ailments is the accumulation of DNA lesions, oxidized proteins, and carbonylated proteins and lipids (Beckman and Ames 1998; Harman 1981; Kujoth *et al.* 2005; Oliver *et al.* 1987; Stadtman 1992). It is well-documented that these defects are caused by reactive molecules such as superoxide anions (O_2_^−^), hydrogen peroxide (H_2_O_2_), and hydroxy radicals (OH) formed as by-products of cellular metabolism (Barrera *et al.* 2008; Bertram and Hass 2008; Shringarpure and Davies 2002; Stadtman 2006).

Less well-studied but no less consequential are the effects of carbonyl stress created by reactive carbonyl compounds (RCC), such as glyoxal, 3DG (3-deoxyglucosone) and methylglyoxal (MG). Glyoxal is formed by lipid and DNA oxidative degradation as well as via autoxidation of glycolaldehyde (Benov and Fridovich 1998). MG is a by-product of metabolic processes, including threonine catabolism (Murata *et al.* 1986) and lipid peroxidation (Poli *et al.* 1985). MG can also arise enzymatically during glycolysis (O’Brien *et al.* 2005; Thornalley 1996). In addition, diverse environmental sources, such as cigarette smoke and automobile exhaust, are abundant sources of carbonyls (O’Brien *et al.* 2005; Saint-Jalm and Moree-Testa 1980; Zervas *et al.* 2002). The widespread thermal processing of food can result in MG and other aldehydes (Nemet *et al.* 2006; Wells-Knecht *et al.* 1995).

Advanced glycation end products (AGE) arising from carbonyl stress are thought to contribute to chronic diseases, such as diabetes, chronic obstructive pulmonary disease, ischemia/reperfusion, and Alzheimer’s disease (Ellis 2007). Understanding the result of carbonyl stress is an essential first step to characterize the impacts of CS stress on cell physiology.

The well-conserved glyoxalase system is the cell’s principal defense against AGEs and aldehydes, detoxifying MG and glyoxal in the presence of glutathione (GSH) to glycolate and D-lactate, respectively (Thornalley 1998). Both can also be detoxified by NADPH-dependent aldose reductases (Vander Jagt *et al.* 1992). Previous work using yeast has focused primarily on MG (Aguilera *et al.* 2005; Maeta *et al.* 2005) and showed that the conserved HOG MAP kinase pathway is important for the induction of MG-responsive genes. However, no comprehensive, genome-wide analysis of the biological consequences of these modifications exists. Identifying the cellular functions necessary for providing resistance to these toxic molecules will provide insight into the molecular mechanisms that underlie diseases associated with CS and could suggest therapeutic interventions.

## Materials and Methods

### Reagents

Methylglyoxal, glyoxal, nicotinamide, isonicotinamide, and aminoguanidine were purchased from Sigma-Aldrich (St. Louis, MO). Nicotinamide and isonicotinamide were dissolved in sterile H_2_O and filtered sterilized.

### Yeast strains, plasmids, and growth conditions

**Figure 1:**
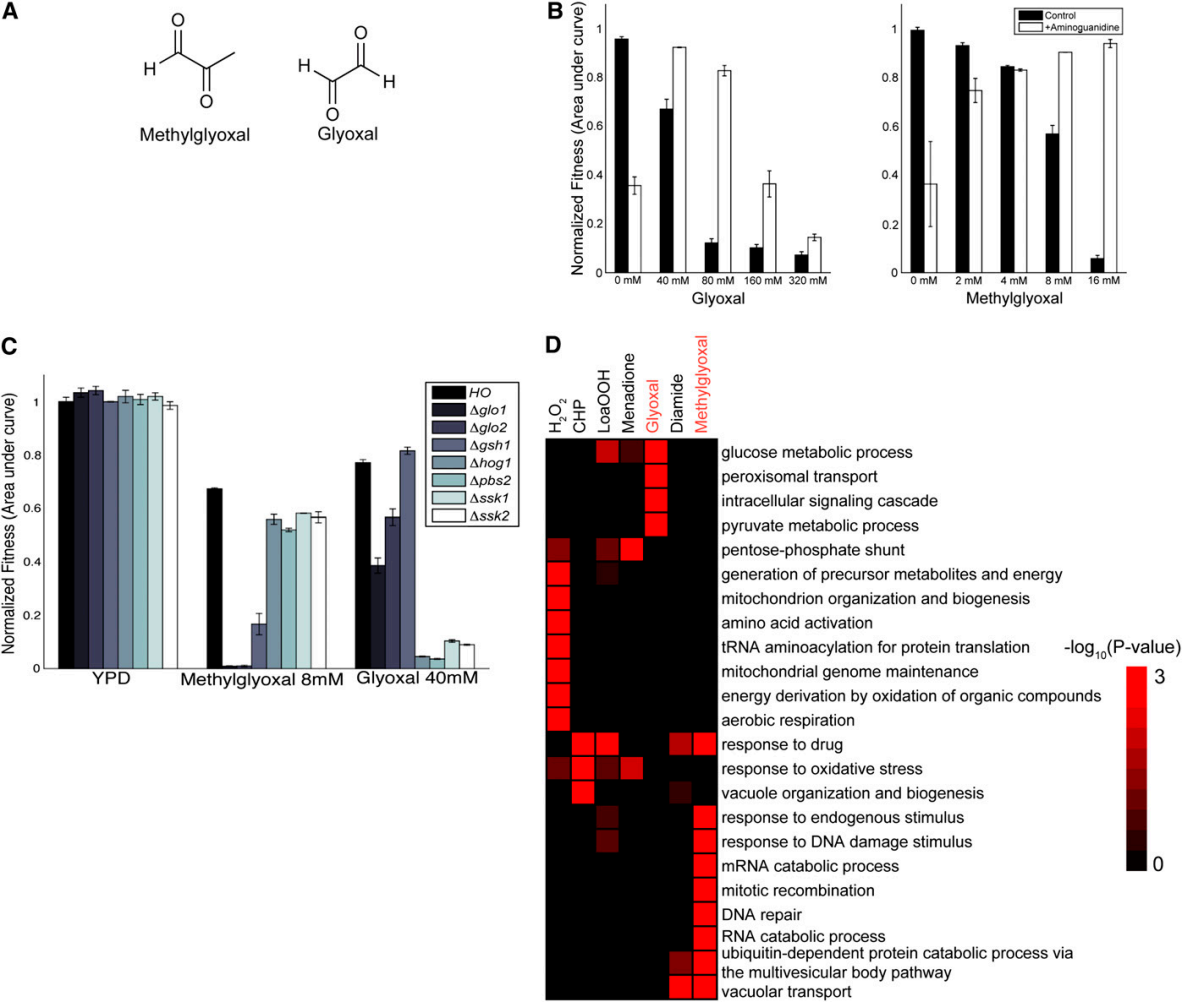
Methylgloxal and glyoxal inhibit yeast growth. A) Chemical structure of methylglyoxal and glyoxal. B) Growth curve analysis for measuring the fitness of wild-type (HO) strain in glyoxal (left) and methylglyoxal (right) in the presence (black bars) and absence of 20 mM aminoguanidine (white bars). Fitness was quantified using area under growth curve and normalized to growth in YPD ± SD (n = 3). Growth inhibition caused by aminoguanidine in the absence of glyoxal and methylglyoxal is discussed in the text. C) Fitness of deletion strains in methylglyoxal and glyoxal ± SD (n = 3) analyzed by growth curve analysis. Strains selected were deficient in genes previously shown to be important for resistance to methylglyoxal stress in yeast. D) Heatmap representing significant GO terms of strains identified to be significantly sensitive (false discovery rate < 0.05) to methylglyoxal and glyoxal by pooled fitness screens. For comparison, we included strains identified to be sensitive to oxidants hydrogen peroxide (H_2_O_2_), cumene hydroperoxide (CHP), linoleic acid 13-hydroperoxide (LoaOOH), menadione, and diamide by a previous study (Thorpe *et al.* 2004). The heatmap is colored according to the *P* value of each GO term.

Yeast deletion strains were obtained from the yeast deletion collection. ORF-containing plasmids, listed in Table S5, were obtained from Charlie Boone or constructed by gap-repair (Oldenburg *et al.* 1997). Yeast transformations were performed using the standard lithium acetate method (Gietz *et al.* 1995) and selected synthetic complete medium lacking uracil (SCM URA−). For growth curve analysis, individual strains were inoculated into 100 μl of YPD or SCM URA− and grown to saturation for ∼20 h at 30°C. Overnight cultures were resuspended by shaking for 15 min, diluted into 100 μl of media in 96-well plates, and grown in Tecan GENios microplate readers for 24 h. The growth rate of each culture was monitored by measuring the OD_600_ every 15 min as previously described (Giaever *et al.* 2004). Doubling-time calculations and area under growth curve (AUGC) analysis were performed as previously described (Hoon *et al.* 2008; Lee *et al.* 2005b). We used AUGC analysis for Figure 1B, as we found that this method more accurately captured the fitness of strains under severe growth inhibition (>50% inhibition). AUGC was calculated uniformly over a 24 h window from the start of the experiment. For lower levels of growth inhibition, doubling time during the exponential growth phase was used for fitness comparisons.

### Genome-wide screening and analysis

MSP and DSP screens were performed as previously described (Hoon *et al.* 2008) with the modification that only the homozygous deletion strains were screened against both methylglyoxal and glyoxal. For DSP, glyoxal was screened at 20 mM and methylglyoxal was screened at 8 mM for 5 and 20 generations. For MSP, yeast genomic library transformants were grown for 20 generations in glyoxal (40 mM) and supplemented with isonicotinamide (25 mM) where indicated. Methods for pooled growth, OD monitoring, automatic cell dilution, and cell harvesting were performed as previously described (Pierce *et al.* 2006). Competitive screens were designed such that strains that are not resistant should get diluted out over the course of four dilutions every 5 generations. There was variation in terms of the number of starting cells for each strain in the pool, but we normalized this by taking the ratio of the microarray intensities between the control and treated pools. Both DSP and MSP were analyzed using a high-density oligonucleotide tag array manufactured by Affymetrix (Pierce *et al.* 2006). For DSP, barcode probe intensities were extracted and processed as previously described (Pierce *et al.* 2006). Each array was mean-normalized and the fold change (log_2_ control/treatment) was calculated by comparison with a set of control arrays. At least two biological replicates were carried out for each treatment condition. The log_2_ ratios of both tags were averaged to generate a single score for each gene for use in the DSP-MSP plots. We used rank product analysis (Breitling *et al.* 2004) to identify strains that were significantly sensitive to either methylglyoxal or glyoxal. A false discovery rate cutoff of 0.05 was used. The list of sensitive strains can be found in Table S1 and Table S2. For the glyoxal deletion resistance screen, the homozygous deletion pools were screened and processed as described above. The results are presented in Table S3. For MSP, ORF probe intensities were extracted and processed in the same way as the barcode probes. Each ORF is represented by at least two probes, and the log_2_ ratios of each probe were averaged to generate a single score for each gene. To identify each suppressor locus, the log_2_ ratio of intensities were ordered by genomic location of each ORF and analyzed using a sliding window to identify loci that have at least two adjacent ORFs with log_2_ ratios ≥ 1.6. GO analysis was performed using GOstats (Falcon and Gentleman 2007), a bioconductor package written in R.

### Double-mutant strain construction

We assessed genetic interactions among a subset of genes that confer resistance to glyoxal by generating multiple combinations of double-deletion strains for quantitative fitness analysis (St. Onge *et al.* 2007). MATa haploids deletion strains were obtained from the yeast deletion collection, and MATα haploids deletion strains were obtained from Charlie Boone. Double-deletion strains were constructed by the synthetic genetic array (SGA) protocol with minor modifications using a Singer RoToR HDA (Singer Instruments, Somerset, UK) (Stansfield and Stark 2007; Tong and Boone 2006). Approximately 800 double-deletion mutants were constructed among 15 deletion strains that were sensitive to glyoxal and 24 deletion strains that were resistant to glyoxal. Single-deletion strains with the same drug resistance cassette (Kan^r^-Nat^r^ and Nat^r^-Kan^r^) as the double-mutant strains were constructed using the HO-deletion strain as a query strain. In MATa haploids, genes were replaced with a kanamycin resistance marker gene (Kan^r^), and in MATα haploids, genes were replaced with a nourseothricin resistance marker gene (Nat^r^). This method allowed each double-deletion gene pair to be constructed twice (Kan^r^-Nat^r^ and Nat^r^-Kan^r^) independently. Fitness values between each gene pair were highly correlated (*R^2^* = 0.92). The fitness of strains was determined in the presence and absence of glyoxal. One double mutant (*Δhog1Δccw12*) could not be constructed because the genes in the pair were genetically linked. Fitness values between each gene pair were highly correlated (Figure 4B, *R^2^* = 0.92), demonstrating the reproducibility of the assay. Strains for which fitness values of Kan^r^-Nat^r^ double-deletion strains differed from that of the Nat^r^- Kan^r^ deletion strains (|W_xy_-W_yx_|/$\sqrt{2}$ > 0.2) were filtered and not used in the analysis.

### Growth assay for epistasis analysis

Deletion strains arrayed on YPD/agar were inoculated into 96-well plates containing 100 μl of YPD using a Singer RoToR HDA (Singer Instruments). Cultures were grown to saturation for 20 h at 30°C and stored at 4°C for 24–48 h. The cells were then resuspended by shaking for 15 min. Cultures were diluted into 100 μl volumes in 96-well plates using the Singer RoToR HDA and grown in Tecan GENios microplate readers for 30 h at 30°C. The growth rate of each culture was monitored by measuring the OD_600_ every 15 min. The doubling time (D) was calculated exactly as previously described (St. Onge *et al.* 2007). The fitness of each deletion strain was calculated as the ratio of the doubling time of the parental wild-type strain divided by that of the mutant. We quantified the genetic interaction between each gene pair using a multiplicative model. If a strain deleted for gene *x* has a fitness of W*_x_* and a strain deleted for gene *y* has a fitness of W*_y_*, then the double mutant strain is expected to have a fitness (W*_xy_*) of W*_x_* × W*_y_*. We measured the deviation ε_xy_ from this expectation, where ε_xy_ = W_xy_ – W_x_ × W_y_. For each gene pair, the ε_xy_ values for each Kan^r^-Nat^r^ and Nat^r^-Kan^r^ were averaged and used to generate the heatmap.

## Results

### Methyglyoxal and glyoxal inhibit yeast growth

MG is a potent inhibitor of yeast growth (Aguilera and Prieto 2001; Aguilera *et al.* 2005; Bito *et al.* 1997; Inoue and Kimura 1996; Inoue *et al.* 1998; Maeta *et al.* 2005). Using a quantitative fitness assay, we quantified the growth inhibition for MG and glyoxal (Figure 1A). The IC_50_ for MG is 8.6 mM (for *S. cerevisiae* strain BY4743), approximately 5-fold less than that for glyoxal (IC_50_ of 45.5 mM) (Figure 1B). Aminoguanidine (AG), a α,β-dicarbonyl scavenging agent (Cameron *et al.* 2005) and known suppressor of carbonyl stress, rescues the growth inhibition caused by both compounds (Figure 1B), suggesting that the cellular effects are specific. In the absence of methylglyoxal or glyoxal treatment with AG (20 mM) inhibits yeast growth, likely due to AG reacting with pyruvate to form a hydrazone adduct at high concentrations (Thornalley 2003); in contrast; the carbonyl compounds modulate this effect.

### HOG pathway components are both required for resistance to MG and glyoxal

In yeast, the high-osmolarity glycerol (HOG) mitogen-activated protein kinase (MAPK) pathway regulates osmotic homeostasis (O’Rourke *et al.* 2002). Previous work has determined that the HOG pathway mediates MG resistance, presumably via promoting the expression of genes involved in MG metabolism (Aguilera *et al.* 2005; Inoue *et al.* 1998). One such gene is *GLO1*, encoding glyoxalase I, an enzyme that converts MG into S-D-lactoylgluthathione in the presence of glutathione (Inoue and Kimura 1996). In contrast, we found glyoxal and MG exert different effects on mutants involved in HOG signaling and glyoxalase activity, suggesting distinct mechanisms of action. We confirmed this with individual growth tests of selected strains in glyoxal and methylglyoxal. For example, strains missing *GLO1*, *GLO2*, or *GSH1* were more sensitive to MG stress than strains missing *HOG1*, *PBS2*, *SSK1*, or *SSK2*, whereas the opposite pattern of sensitivities was observed for glyoxal stress (Figure 1C).

### Genome-wide fitness profiling demonstrates physiologically distinct responses to MG and glyoxal

We used two genome-wide assays: 1) yeast deletion collection to identify genes that confer sensitivity, and 2) an overexpression assay to define genes that confer resistance to CS. Genome-wide fitness profiling showed that glyoxal and MG affect cells very differently. We screened both compounds against a collection of 4700 homozygous deletion strains (Giaever *et al.* 2002; Giaever *et al.* 2004; Hillenmeyer *et al.* 2008; Hoon *et al.* 2008; Lee *et al.* 2005b) and identified 458 deletion strains that were significantly sensitive (false discovery rate < 0.05) to either MG or GLY (supporting information, Table S1 and Table S2). Functional enrichment analysis using Gene Ontology (GO) annotations of sensitive strains showed distinct requirements for resistance to each compound (Figure 1D). MG-sensitive genes were enriched for protein, mRNA, and DNA metabolic processes, in agreement with the suggested mechanism of action of MG in forming DNA, RNA, and protein adducts (Kalapos 1999). In contrast, glyoxal resistance required genes involved in glucose metabolism and peroxisomal and signal transduction processes. Our study corroborates, on a genome-wide level, previous studies in which MG and glyoxal were shown to exert different effects *in vivo* [*e.g.*, MG and glyoxal induced distinct signals for MAP family kinases in human endothelial cells (Akhand *et al.* 2001)].

The effects of MG and glyoxal were also distinct from that observed for other oxidants. We compared GO annotations for genes identified following treatment with other oxidants in a previous study (Thorpe *et al.* 2004) and found that the effects of oxidative stress are typically broad but specific to each oxidant (Figure 1D). Here, we found that additional functions, beyond those associated with ROS, were important for CS resistance.

### Carbonyl stress leads to DNA, RNA, and protein dysfunction

We found that multiple processes involved in the repair and degradation of damaged cellular macromolecules factor in the CS response. Glyoxal and MG are potent arginine-directed glycating agents and covalently cross-link proteins via modification of arginine residues, leading to protein dysfunction (Rabbani and Thornalley 2008). Protein modification by MG activates ubiquitin/proteasome-dependent proteolysis (Du *et al.* 2006). Consistent with this observation was our finding that strains deleted for genes involved in ubiquitin-dependent protein degradation (∆*shp1*, ∆*bsd2*, ∆*stp22*, ∆*doa4*, ∆*swm1*, ∆*bst1*, ∆*cdc26*, ∆*rad6*, ∆*eps1*, ∆ *rtt101*, ∆*grr1*, ∆*vps25*, ∆*def1*, ∆*doa1*, ∆ *snf7*, ∆ *srn2*, ∆*vps36*, ∆*ubx2*, ∆*ubx4*, ∆*vps20*, ∆*ydj1*, ∆*snf8*, ∆*vps28*, and ∆*bro1*) were sensitive to MG. For glyoxal, the requirement for ubiquitin-related pathways was less pronounced, with fewer identified strains (∆*reg1*, ∆*doa4*, ∆*ubc8*, ∆ *jem1*, and ∆*ydj1*). Together, the MG and GLY results demonstrate that protein catabolism plays a major role in detoxifying glycated proteins.

Besides producing protein adducts, methylglyoxal also reacts with guanine bases in RNA and DNA (Kang *et al.* 1996; Murata-Kamiya and Kamiya 2001). Because messenger RNA (mRNA) quality control is essential, we expected to uncover gene deletion strains involved in mRNA metabolism. The first step in mRNA decay is deadenylation, followed by mRNA degradation either by decapping followed by 5′→3′ decay or by 3′→5′ decay (Garneau *et al.* 2007). As expected, several strains deficient in deadenylation (∆*ccr4*, ∆*pop2*, and ∆*not5*), decapping and 5′→3′ decay (∆*dhh1*,∆*pat1*,∆*lsm6*, ∆*lsm1*, and ∆*lsm7*) and 3′→5′ decay (∆*ski8*, ∆*ski7*, ∆*ski3*, and ∆*ski2*) were sensitive to methylglyoxal. Furthermore, multiple strains involved in DNA repair (∆*mms4*, ∆*rad18*, ∆*rpn4*, ∆*rad59*, ∆*rad57*, ∆*rad55*, ∆*hpr1*, ∆*mus81*, ∆*rad51*, ∆*rad4*, ∆*rad6*, ∆*rpb9*, ∆*rad54*, ∆*srs2*, ∆*def1*, ∆*doa1*, ∆*rad5*, ∆*mms22*, ∆*rad52*, ∆*rad14*, ∆*mre11*, ∆*tho2*, ∆*rad50*, ∆*snf2*, ∆*rad1*, and ∆*mms1*) were also sensitive to MG. In contrast, DNA repair and mRNA decay pathways were not required for glyoxal resistance, even at higher doses (data not shown). Together, these results demonstrate that MG damages cellular macromolecules more potently than glyoxal.

### Glyoxal-resistant deletion strains

**Figure 2:**
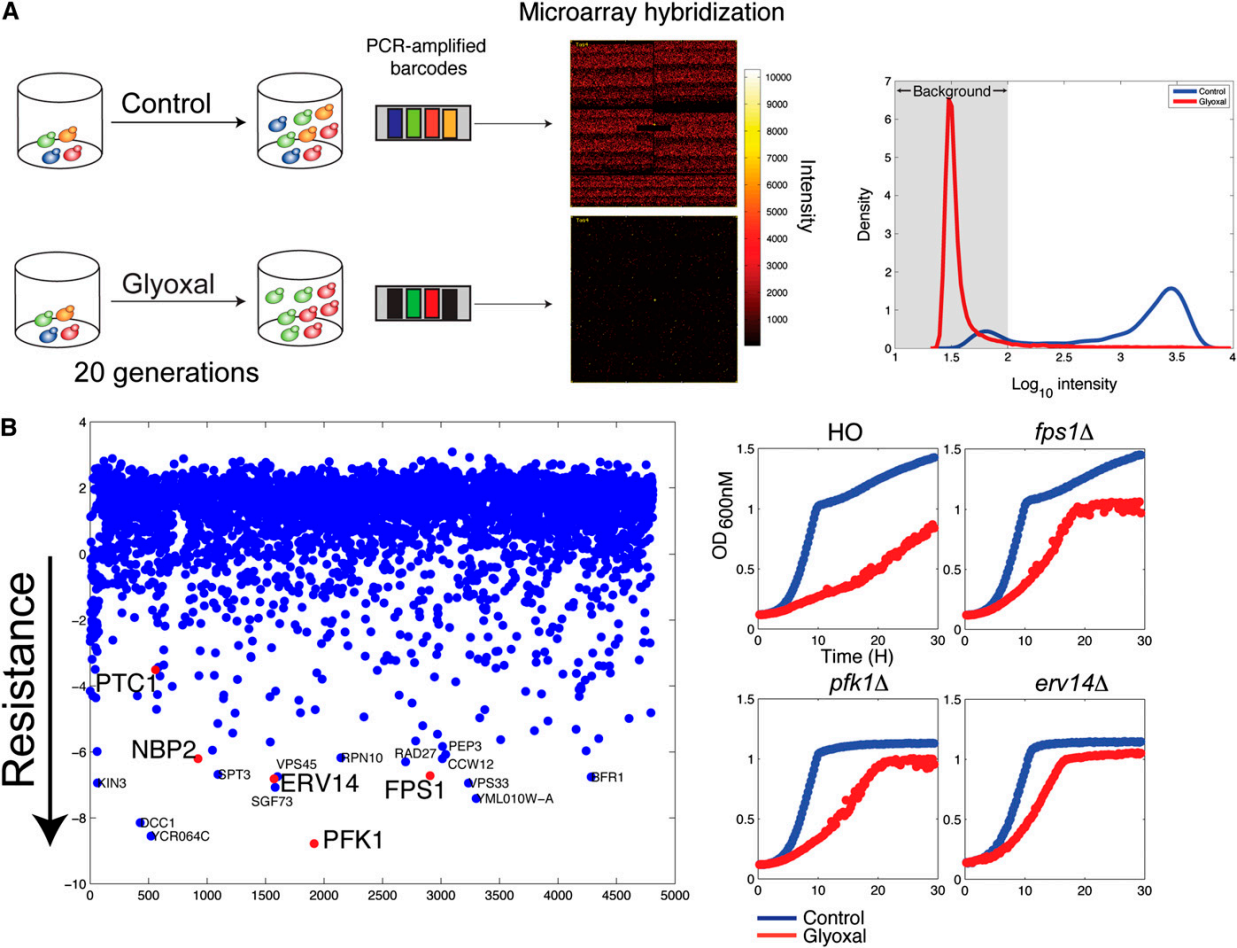
Identifying deletion strains resistant to glyoxal stress. A) (left) Schematic of screen used for identifying deletion strains resistant to glyoxal stress. A homozygous deletion pool was grown for 20 generations in 80 mM glyoxal. Barcodes were amplified and hybridized to TAG4 arrays. (right) Strong selection in the presence of glyoxal resulted in selection for strains highly resistant to glyoxal. TAG intensities are mostly at background (gray region) in cells treated with glyoxal compared to control. B) (left) Log_2_ fold ratio of control/treatment of TAG array results. Negative fold ratios indicate tags that are over-represented in glyoxal selection. Genes mentioned in the text are highlighted in red. (right) Confirmation growth curves of deletion strains resistant to glyoxal.

Having identified numerous CS-sensitive deletion strains, we asked which deletion strains manifest CS resistance. Such genes can be interpreted as negative regulators of stress resistance. Pooled fitness profiling assays have focused on identifying deletion strains with reduced fitness in experimental conditions, in part because the assay’s dynamic range for assessing resistance is poor [*i.e.*, modest perturbations are used (∼IC_10_)] and in part because a large amount of the sample is hybridized. To identify resistant strains, we developed an optimized “resistance screen” in which the entire pool of deletion strains is treated with a high dose of compound (*e.g.*, IC_50_) over 20 generations of growth (Figure 2A and *Materials and Methods*), and a smaller amount of material is hybridized (Figure 2A). This new assay identified *bona fide* resistant deletion strains. Following treatment with glyoxal at an IC_50_ concentration, most of the strain-specific TAG intensities were at background levels compared to controls, while those strains that remained constant or increased during the course of the experiment represented glyoxal-resistant strains.

Multiple strains were significantly resistant to glyoxal, and the majority of these strains were confirmed individually (Figure 2B, Table S3). Among these genes, *PTC1*, a type 2C protein phosphatase that negatively regulates the HOG pathway by dephosphorylating Hog1 (Warmka *et al.* 2001), and Nbp2, a protein that recruits Ptc1 to the Pbs2-Hog1 complex, provide additional evidence that upregulation of the HOG pathway is required for glyoxal resistance (Mapes and Ota 2004). GO analysis of these resistant deletion strains showed enrichment for intracellular/endosome transport protein metabolic processes and organelle organization (Table S4**)**.

### A sensitized suppressor screen to understand glyoxal stress

To further characterize the effects of glyoxal, we employed a third genome-wide assay. Specifically, we identified multicopy suppressors of glyoxal sensitivity using multicopy suppressor profiling (MSP) to identify genes that confer resistance to compounds when over-represented (Hoon *et al.* 2008; Rine *et al.* 1983).

**Figure 3:**
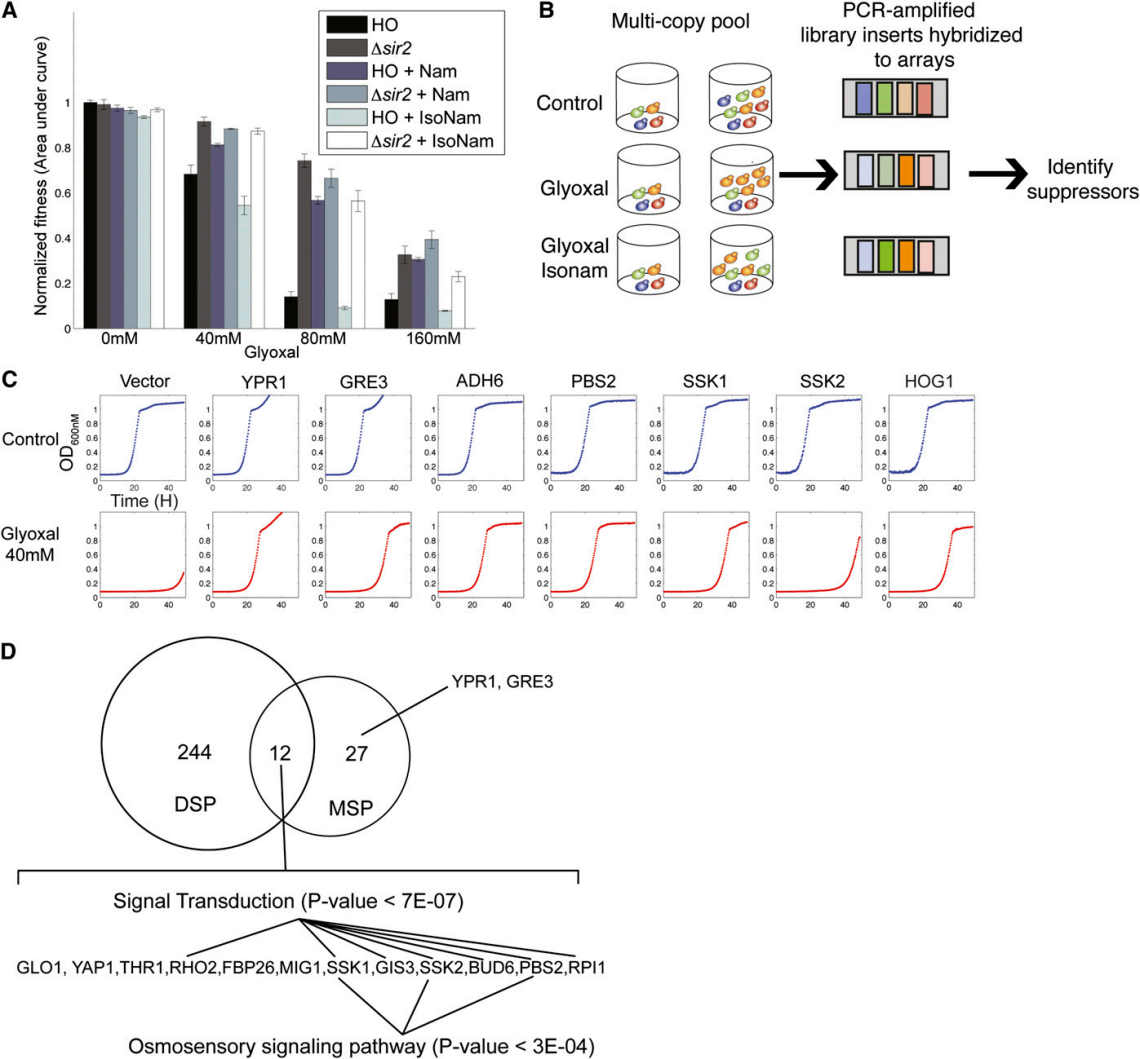
Sensitized glyoxal suppressor screen. A) Fitness ± SD (n = 3) of wild-type and *SIR2* mutants grown in different glyoxal concentrations with and without supplementing nicotinamide (NAM) and isonicotinamide (IsoNAM) at 5 mM and 25 mM, respectively. B) Schematic of multicopy suppressor screen with glyoxal and isonicotinamide. A pool of yeast strains harboring a genomic library was grown competitively in the presence of control, glyoxal, and glyoxal supplemented with isonicotinamide. Plasmids were isolated and inserts were amplified by PCR and hybridized to ORF probes present on the TAG4 array. Over-represented ORFs in treatment *vs.* control are identified as candidate suppressors. C) Confirmatory growth curves of individually cloned suppressors identified by MSP. D) Overlap of genes identified by MSP and DSP. MSP genes include genes found in suppressor loci in Table 1. In cases where individual genes were not identified by subcloning, all genes in the suppressor loci were included. Genes that were found in both assays were enriched for components in the osmosensory signaling pathway by Gene Ontology (GO) analysis.

During our experiments with deletion resistant screens, we discovered that *sir2* deletion mutants displayed resistance to glyoxal. This observation, combined with the well-characterized relationship between Sir2 and NAD metabolism (Blander and Guarente 2004), inspired us to test if nicotinamide, an endogenous inhibitor of Sir2 (Bitterman *et al.* 2002), would act as a chemical modifier in the glyoxal MSP assay. We found that nicotinamide conferred resistance to glyoxal (Figure 3A) whereas isonicotinamide, an antagonist of nicotinamide inhibition and an activator of Sir2 deacetylase activity (Sauve and Schramm 2004), conferred sensitivity to glyoxal (Figure 3A). Therefore, we mimicked activation of Sir2 using isonicotinamide to amplify the sensitivity of the MSP screen (Figure 3B). We identified multiple suppressors containing genomic DNA that were over-represented in pools grown in glyoxal compared with control (Table 1), and isonicotinamide administration identified additional suppressors. One of the most resistant loci identified was *GLO1*, the enzyme that metabolizes glyoxal. This observation confirms that this experimental approach can identify relevant genes involved in glyoxal resistance. Several suppressors were confirmed by isogenic tests with wild-type yeast harboring plasmids containing individually cloned ORFs (Figure S1).

**Table 1:** Multicopy suppressors identified by MSP

Locus	Log_2_ Ratio	Average Ratio	Confirmed Suppressor
Glyoxal			
* GLO1;YML003W*	2.52,3.25	2.88	*GLO1*
* YDR366C;YDR367W;YPR1;XRS2*	3.29,2.07,2.20,2.37	2.48	*YPR1*
* YMR317W;ADH6*	2.86,2.04	2.45	*ADH6*
* PBN1;LRE1*	2.13,2.35	2.24	ND
Glyoxal + isonicotinamide			
* PBN1;LRE1*	4.81,4.74	4.78	ND
* YDR366C;YDR367W;YPR1;XRS2*	5.05,4.14,4.53,4.73	4.61	*YPR1*
* YMR317W;ADH6*	4.73,4.49	4.61	*ADH6*
* MRPL28;STP1*	3.07,4.24	3.66	*STP1*
* MED8;SOY1;MSI1;PGI1*	4.25,4.09,3.68,2.60	3.65	*MED8*
* YML007C-A;YAP1;GIS4;[tS(AGA)M];TRM12;GLO1;YML003W*	2.40,2.46,2.33,0.00,4.90,5.80,6.43	3.47	*GLO1*
* THR1;PPA1;RPN1*	3.16,3.83,3.11	3.37	*THR1*
* ZDS2;YML108W;PML39*	3.60,3.48,2.86	3.31	*ZDS2*
* NST1;RHO2*	2.88,3.58	3.23	*RHO2*
* YGR125W;YGR126W*	2.42,3.66	3.04	*YGR126W*
* FBP26;VPS35*	2.95,2.96	2.96	ND
* CCC2;[YDR271C];GLO2;DON1*	2.94,0.00,4.38,4.04	2.84	*GLO2*
* YGL036W;MIG1*	3.38,2.26	2.82	ND
* LCP5;YER128W*	2.94,2.65	2.79	ND
* SSL1;SSK1*	2.50,3.09	2.79	*SSK1*
* MIG2;SIP2*	2.56,3.00	2.78	*MIG2*
* GIS3;IOC2*	2.14,3.39	2.77	*GIS3*
* BUD22;ERG5;SOK2*	2.40,2.76,2.91	2.69	ND
* STB1;KRI1*	2.91,2.40	2.66	ND
* YKR023W;DBP7*	2.73,2.45	2.59	ND
* TRR2;CDC12*	2.55,2.52	2.54	ND
* NIP7;SRP72*	2.57,2.44	2.50	ND
* ALG12;SSK2*	2.00,2.79	2.40	ND
* RBA50;[snR84];HLR1;QCR7;APA2*	3.36,0.00,3.30,3.04,2.19	2.38	*HLR1*
* YGR016W;YGR017W*	2.42,2.18	2.30	ND
* BSP1;YPR172W;VPS4*	2.14,2.38,2.39	2.30	ND
* DDP1;YOR164C*	2.27,2.29	2.28	ND
* CBS1;[YDL068W];COX9*	2.00,0.00,2.48	1.49	ND
Singletons			
* DAM1*	3.55		ND
* MTH1*	3.10		ND
* CDC34*	3.00		ND
* BUD6*	2.90		ND
* PBS2*	2.90		*PBS2*
* RPI1*	2.87		ND
* SEC6*	2.85		ND
* YPT7*	2.83		ND
* DNA2*	2.82		ND
* GRE3*	2.70		*GRE3*

Syntenic genes identified by MSP screen. Singletons are genes that do not have neighboring genes with high log_2_ ratios. ORFs in brackets [] are not present on the microarray and thus not assessed by MSP. ND, not tested by single ORF analysis.

### Deletion- and overexpression-based assays are complementary

Surprisingly, only 4% (12/287) of genes were identified in both the overexpression-resistance and deletion-sensitivity assays (Figure 3D). There are multiple reasons for this lack of overlap, both biological and technical. These assays were designed to yield orthologous data; *e.g.*, deletion screens are unlikely to identify genes with overlapping or redundant function (Tong *et al.* 2001). Overexpression screens can identify suppressor genes that are functionally redundant because expression of a single gene can confer resistance. Indeed, we identified several aldehyde reductases that, when overexpressed, behaved this way. *GRE3* is an aldehyde reductase that is capable of metabolizing MG and is regulated by the HOG pathway (Aguilera and Prieto 2001; Aguilera *et al.* 2005). *YPR1* is another aldehyde reductase that has high specificity for 2-methylbutyraldehyde (Ford and Ellis 2002). *ADH6* is an alcohol dehydrogenase able to detoxify aldehydes *in vivo* (Larroy *et al.* 2002). All three genes strongly suppressed glyoxal toxicity in single-strain confirmations (Figure 3C). Although each of these genes was identified by overexpression profiling, none was sensitive to glyoxal as a single-gene deletion (Table S2), reflecting potential functional redundancy and underscoring the benefit of combining the results of multiple assays.

Despite the small overlap between loss-of-function and gain-of-function assays, 12 genes where identified in both assays (Figure 3D). These genes were enriched for signal transduction; *e.g.*, three components of the HOG pathway (*PBS2*, *SSK1*, and *SSK2*) were identified. Overexpression of *HOG1* conferred resistance to glyoxal (Figure 3C), even though it was not identified by MSP. On the basis of our earlier work, genes that ranked high in both assays were enriched for functions directly related to drug mechanism of action (Hoon *et al.* 2008); accordingly, we concluded that the HOG pathway is critical for glyoxal resistance, and because mutants in the glyoxalase pathway were much less sensitive (Figure 1C), we suggest that additional effectors of glyoxal resistance are regulated by the HOG pathway.

### Integrating genome-wide glyoxal sensitivity and resistance data

**Figure 4:**
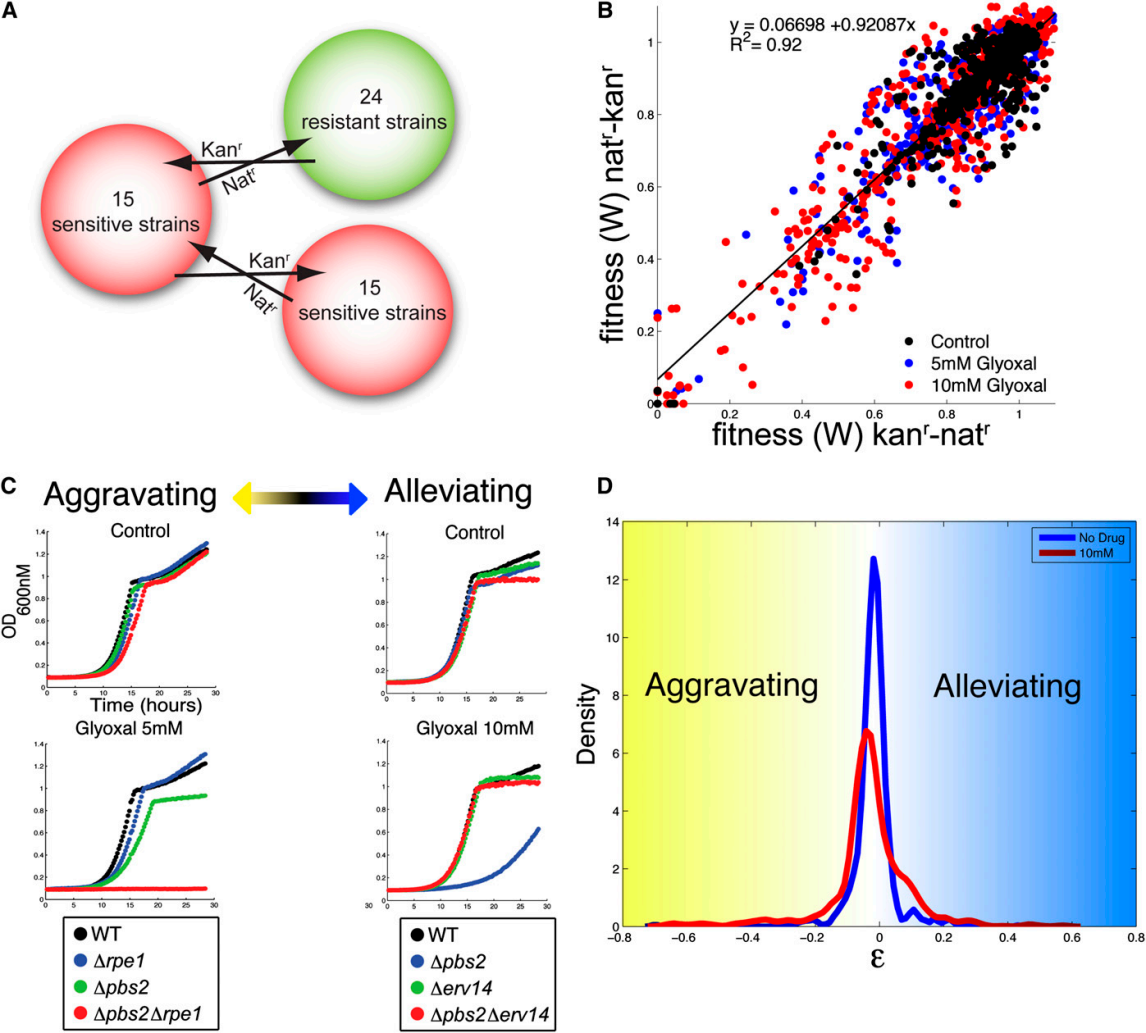
Quantitative epistasis analysis. A) Double deletion strains were constructed between 15 glyoxal sensitive strains and 24 glyoxal resistant strains. Each double mutant was constructed independently twice using two different markers (Kan^r^ and Nat^r^). B) Fitness correlation between reciprocal double-deletion mutants for each gene pair in the presence and absence of glyoxal. The correlation coefficient (R) and the best fitting line are shown. C) Examples of double-deletion mutants displaying aggravating and alleviating genetic interaction in the presence of glyoxal. Δ*pbs2*Δ*rpe1* mutants were strongly aggravating in 5 mM glyoxal with ε = −0.66, and Δ*pbs2*Δ*erv14* were strongly alleviating (suppression) in 10 mM glyoxal with ε = 0.44. D) Distribution of ε values for all double mutant pairs grown in YPD (blue) or 10 mM glyoxal (red).

Using the data from the deletion- and overexpression-based screens, we generated a ranked list of sensitive and resistant strains and performed epistasis analysis by systematically creating double mutants between genes involved in CS resistance and measuring their fitness in the presence of glyoxal using high resolution growth curve analysis on the approximately 800 multimutants. Epistasis, defined as the influence of a mutation in one gene on the phenotype of another, can be formalized so that any deviation from the predicted effects of combined mutations can be quantified to derive genetic interaction networks and dissect cellular pathways (Boone *et al.* 2007; Mani *et al.* 2008) (St. Onge *et al.* 2007). All possible combinations of double-deletion strains were constructed for 15 deletion strains sensitive to glyoxal, and all possible combinations between the same 15 sensitive deletion strains and 24 resistant deletion strains were constructed for a total of ∼800 double-deletions strains that were screened for fitness in the presence and absence of glyoxal (Figure 4A).We performed the screens at two glyoxal concentrations (5 mM and 10 mM) that were lower than the pooled screen concentration to increase the likelihood of identifying both alleviating and aggravating interactions. Each double mutant was constructed twice, and we found that fitness values between reciprocal gene pairs were highly correlated (Figure 4B). Using fitness values for double- and single-deletion strains, we quantified the genetic interaction (expressed as ε) between gene pairs, where ε_xy_ = W_xy_ – W_x_ × W_y_. By performing our screens at multiple drug doses with both resistant and sensitive mutant strains (using high-resolution growth curves), we could identify subtle genetic interactions ranging from aggravation (ε < 0) to suppression (ε > 0). For example, in 5 mM glyoxal, loss of *RPE1* severely aggravated glyoxal sensitivity of a Δ*pbs2* strain, whereas in 10 mM glyoxal, loss of *ERV14* completely suppressed glyoxal sensitivity of a Δ*pbs2* strain (Figure 4C). The distribution of ε values showed that a greater number of genetic interactions were uncovered when the screen was performed in the presence of stress (St. Onge *et al.* 2007) (Figure 4D).

**Figure 5:**
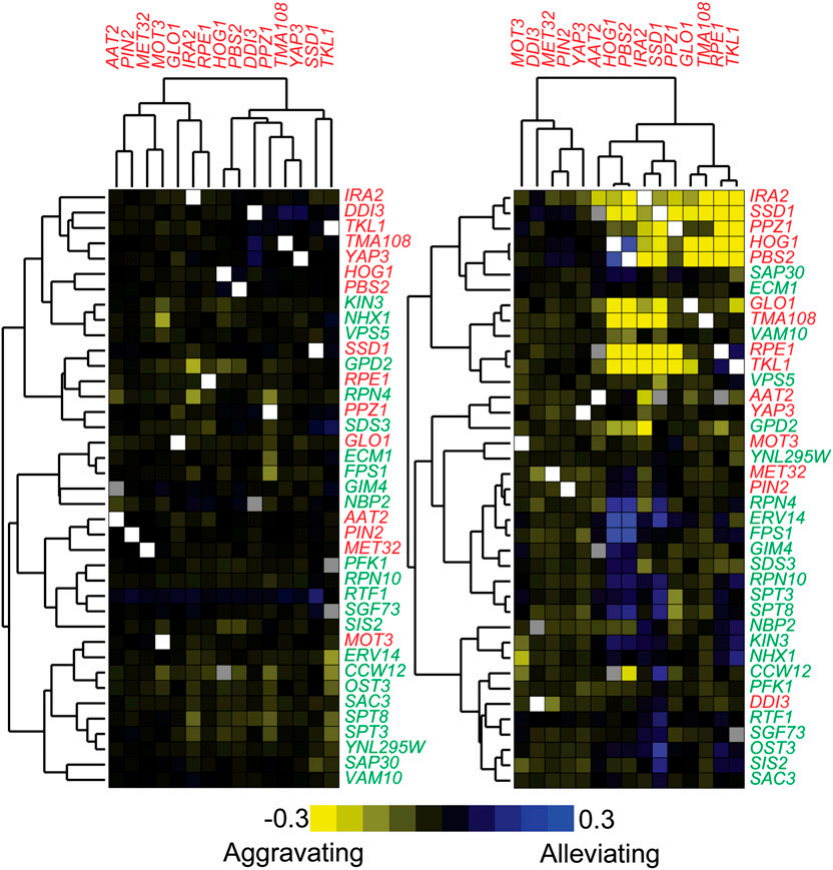
Genetic interaction profiles predict pathways. Genes hierarchically clustered (Pearson correlation) according to similar patterns of genetic interaction (ε) for growth in YPD (left) and in 10 mM glyoxal (right). The heatmap is colored accordingly to the ε value of each double mutant: yellow for aggravating interactions (ε < 0) and blue for alleviating interactions (ε > 0). Genes identified as sensitive to glyoxal when deleted singly are highlighted in red, and genes identified as resistant to glyoxal when deleted singly are highlighted in green.

We summarized all the epistatic tests as a heatmap, with strains ordered by hierarchical clustering of their ε values (Figure 5). Genes of related function clustered together; *e.g.*, *HOG1* and *PBS2* were highly correlated (r = 0.98) in both YPD and YPD + glyoxal. In contrast, other pairs of genes shared genetic interactions only in the presence of glyoxal. *RPN10*, which encodes the 19S regulatory particle (RP) of the 26S proteasome, clusters with *SPT3* and *SPT8*, members of the SAGA transcriptional regulatory complex. It was recently shown that the 19S RP alters SAGA and stimulates its interaction with transcription activators (Lee *et al.* 2005a). We also note that a strain deleted for *GCN5*, the catalytic subunit of SAGA, was resistant to glyoxal (Table S3) and *RPE1*, an epimerase with roles in the non-oxidative part of the pentose phosphate pathway that has been shown to display increased sensitivity to hydrogen peroxide (Juhnke *et al.* 1996). In the presence of glyoxal, *RPE1* clusters with *TKL1*, a transketolase that functions in the same pathway (Walfridsson *et al.* 1995), and both are involved in NADPH production, important for protection against reactive oxidative stress. Because alleviating interactions arise when a mutation in one gene impairs the function of a pathway, thereby masking the effects of mutations in other members of the same pathway (Mani *et al.* 2008), the alleviation observed between *RPE1* and *TKL1* (ε = 0.15) suggests that the two genes operate in the same pathway. In contrast, mutants of both *RPE1* and *TKL1* shared aggravating interactions with mutants of *IRA2*, *SSD1*, *PPZ1*, *HOG1*, and *PBS2*, indicating that they buffer glyoxal resistance via distinct pathways. Thus, our epistasis analysis of over 800 multimutants detected known relationships between genes in the same pathway and uncovered novel relationships between pathways that were detected only under glyoxal-induced stress.

### Loss of Fps1 and Erv14 abolishes the requirement for the HOG pathway

**Figure 6:**
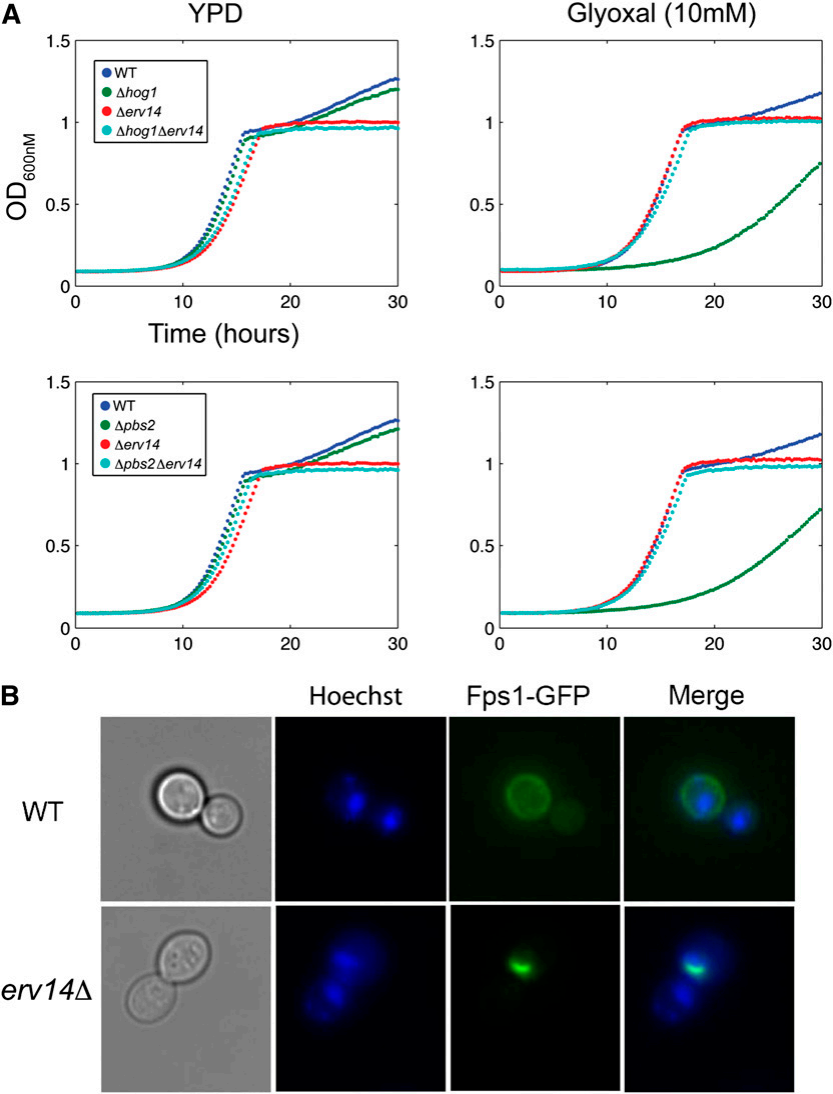
Erv14 deletion abolishes requirement for HOG pathway. A) Growth curve of double mutants Δ*hog1*Δ*erv14* (top row) and Δ*pbs2*Δ*erv14* (bottom row) in YPD and glyoxal (10 mM). B) Live cell imaging of Fps1 distribution. Wild-type (BY4741) and Δ*erv14* cells expressing C-terminally GFP-tagged Fps1 from the pUG23-FPS1GFP plasmid were grown in synthetic media lacking methionine. DNA staining was performed with Hoescht stain 33258.

Erv14 is a transmembrane protein found in the ER and the early Golgi compartment involved in COPII cargo selection (Nakanishi *et al.* 2007; Powers and Barlowe 1998; Powers and Barlowe 2002). Our genetic interaction analysis showed that Erv14 clusters with Fps1, a membrane channel involved in glycerol export. Deletion of both genes resulted in complete suppression of glyoxal sensitivity of Δ*hog1* and Δ*pbs2* mutants, demonstrating that both *FPS1* and *ERV14* function downstream of the HOG pathway (Figure 6A). Fps1 is known to mediate the uptake of acetic acid, arsenite, and antimonite, and downregulation of Fps1 activity via Hog1 confers resistance to the same toxins (Mollapour and Piper 2007; Thorsen *et al.* 2006; Wysocki *et al.* 2001). On the basis of the degree of resistance conferred by loss of *FPS1* to both Δ*hog1* and Δ*pbs2* mutants, we speculate that glyoxal may enter the cell via the Fps1 plasma membrane channel. Because loss of Fps1 also suppressed glyoxal sensitivity in other mutants (*e.g.*, Δ*tma108*, Δ*tkl1*, and Δ*ppz1*) at much higher glyoxal concentrations (Figure S2), it is also possible that Erv14 regulates transport of Fps1 to the plasma membrane. To test the hypothesis that loss of Erv14 renders cells resistant to glyoxal by disrupting plasma membrane localization of Fps1, we examined a GFP-tagged allele of Fps1 and found that, in fact, localization of Fps1 to the plasma membrane was disrupted in Δ*erv14* mutants (Figure 6B). This observation confirmed that epistatic studies derived from the genome-wide screens can form the basis of specific, testable hypotheses.

## Discussion

Characterization of chemical stresses, such as MG and glyoxal, is challenging because numerous pathways (*e.g.*, stress response and chemical detoxification) are involved. To address these challenges, we employed several unbiased chemical genomic assays. First, using competitive fitness measurements, we defined the genetic determinants of CS resistance and then determined the relationships between these genes in the presence of CS using multimutant analysis (St. Onge *et al.* 2007). The result is a comprehensive view of the physiology that underlies the cellular response to CS. We show that the genetic requirements for MG and glyoxal resistance are distinct and that different carbonyl stresses require distinct genetic cohorts for cell survival. We demonstrate the importance of macromolecule catabolism for resistance to MG, confirming and extending known mechanisms of MG toxicity. Downregulation of the glucose repression pathway was also implicated in the control of glyoxal resistance, specifically *MIG1*, *SNF1*, and *MIG2* (Table 1).

**Figure 7:**
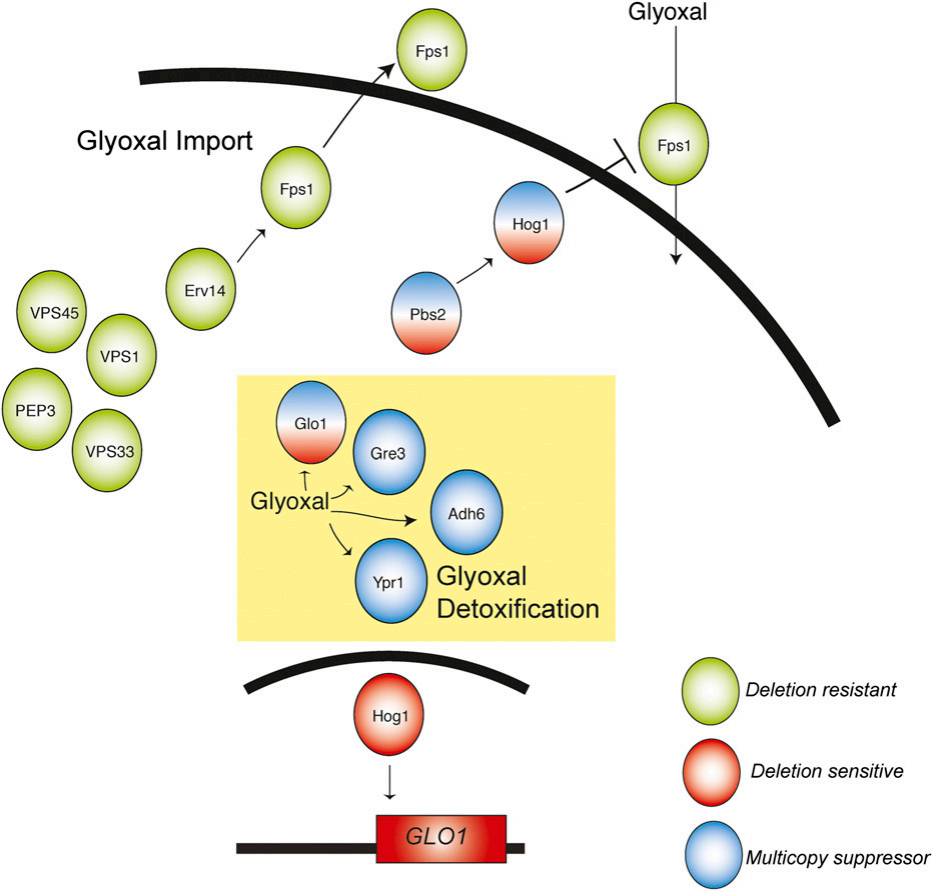
Model for glyoxal resistance. We highlight four main pathways necessary for glyoxal resistance. The HOG pathway positively regulates the expression of *GLO1* and negatively regulates *FPS1* in conferring glyoxal resistance. We hypothesize that the HOG pathway targets Fps1 for degradation, thereby reducing glyoxal import. Moreover, we hypothesize that transport of Fps1 to the cell surface is mediated by Erv14 and associated proteins. We also identified two other mechanisms by which glyoxal resistance is managed: 1) downregulation of the Ras/cAMP/PKA pathway and 2) glyoxal metabolism.

The effects of glyoxal on the cell are pervasive and deleterious, yet poorly understood. Integrating our observations, we suggest a model of glyoxal resistance in yeast (Figure 7) with the intention of generating testable hypotheses for follow-up studies. In this model, glyoxal detoxification is mediated via multiple reductases, *GRE3*, *ADH6*, *YPR1* and *GLO1*, which were identified via multicopy suppression to confer glyoxal resistance. A functioning HOG pathway is critical for glyoxal resistance by regulating the expression of *GLO1*, which encodes a glyoxal detoxifying enzyme. In addition to *GLO1*, our data suggest that other downstream effectors participate in mediating glyoxal resistance. One candidate effector is *FPS1*, a plasma membrane channel that is targeted for endocytic degradation by Hog1 phosphorylation (Mollapour and Piper 2007). Deletion of *FPS1* abolishes the sensitivity of HOG pathway mutants to glyoxal, similar to that observed for acetic acid, arsenite, and antimonite (Mollapour and Piper 2007; Thorsen *et al.* 2006; Wysocki *et al.* 2001). Several mutants responsible for ER and early Golgi transport were observed to be resistant to glyoxal. In particular, loss of *ERV14* also abolishes sensitivity of HOG pathway mutants. We speculate that this is due to defective localization of Fps1 to the cell surface with a concomitant reduction in glyoxal levels. One way to test this model would be to measure the intracellular glyoxal concentrations during treatment of wild-type and mutant strains.

In summary, we show the broad range of specific cellular functions that are important for carbonyl stress resistance. Using multiple genome-wide approaches, we were able to comprehensively identify multiple genetic requirements for glyoxal stress resistance. Furthermore, a quantitative epistasis analysis comprising ∼800 strains allowed us to uncover glyoxal-dependent genetic interactions. These data comprise the first genome-wide assessment of the role of gene dose and of epistatic relationships with respect to their role in the response to carbonyl stress. Because many of the pathways affected by carbonyl stress are well conserved, our observations and their underlying datasets provide a resource for future studies in yeast and other cell types. For example, aging, while not typically thought of as a stress-related disorder, manifests many of the features of carbonyl accumulation–related toxicity. Accordingly, comprehensive gene dose studies may serve as a cost-effective and comprehensive approach for toxicity assessment toward achieving the goal of transforming both general and environmental health (Collins *et al.* 2008).

## Supplementary Material

Supporting Information
