# Supplementary material for: A Global Perspective of the Genetic Basis for Carbonyl Stress Resistance
Source: G3 (Bethesda). 2011 Aug 1;1(3):219–31. doi: 10.1534/g3.111.000505 (PMC3276133; doi:10.1534/g3.111.000505)
Supplement: Supporting Information [file supp_1_3_219__index.html]

Supporting Information 

# A Global Perspective of the Genetic Basis for Carbonyl Stress Resistance

## Supporting Information for Hoon *et al.*, 2011

**Files in this Data Supplement:**

- Supporting Information - Figure S1 and S2 and Tables S1-S5 (PDF, 544 KB)
- Figure S1 - Confirmation growth curves of multicopy suppressors identified from MSP screen with glyoxal and/or isonicotinamide (PDF, 148 KB)
- Figure S2 - Loss of *Δfps1* suppresses glyoxal sensitivity of *Δtma108*, *Δtkl1*, *Δppz1*, *Δpbs2*, *Δhog1* mutants at higher glyoxal concentrations (PDF, 276 KB)
- Table S5 - Plasmids used in this study (PDF, 88 KB)
- Table S1 - Strains that are significantly sensitive to methylglyoxal (false discovery rate < 0.05) (Microsoft Excel, .xls, 112 KB)
- Table S2 - Strains that are significantly sensitive to glyoxal (false discovery rate < 0.05) (Microsoft Excel, .xls, 104 KB)
- Table S3 - log2 ratio values for the glyoxal resistance screen for three replicates performed on the homozygous deletion pool (Microsoft Excel, .xls, 1.4 MB)
- Table S4 - Gene Ontology enrichment for the deletion strains resistant to glyoxal as determined by GO TermFinder (Microsoft Excel, .xls, 28 KB)
