# Supplementary material for: A Global Perspective of the Genetic Basis for Carbonyl Stress Resistance
Source: G3 (Bethesda). 2011 Aug 1;1(3):219–31. doi: 10.1534/g3.111.000505 (PMC3276133; doi:10.1534/g3.111.000505)
Supplement: Supporting Information [file supp_1.3.219_FigureS1.pdf]

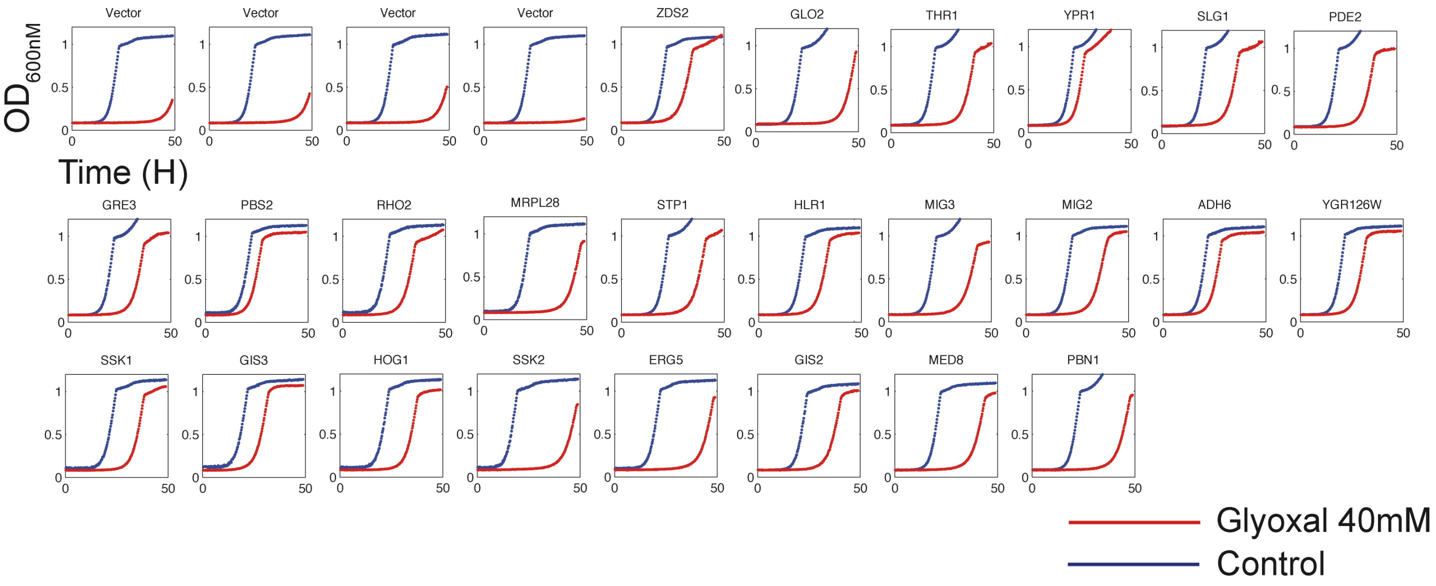

Figure S1 Confirmation growth curves of multicopy suppressors identified from MSP screen with glyoxal and/or isonicotinamide. Singly cloned ORFs under the control of native promoters on 2μ plasmids were grown in Leu-synthetic media in the presence or absence of glyoxal.
