# Supplementary material for: A Global Perspective of the Genetic Basis for Carbonyl Stress Resistance
Source: G3 (Bethesda). 2011 Aug 1;1(3):219–31. doi: 10.1534/g3.111.000505 (PMC3276133; doi:10.1534/g3.111.000505)
Supplement: Supporting Information [file supp_1.3.219_FigureS2.pdf]

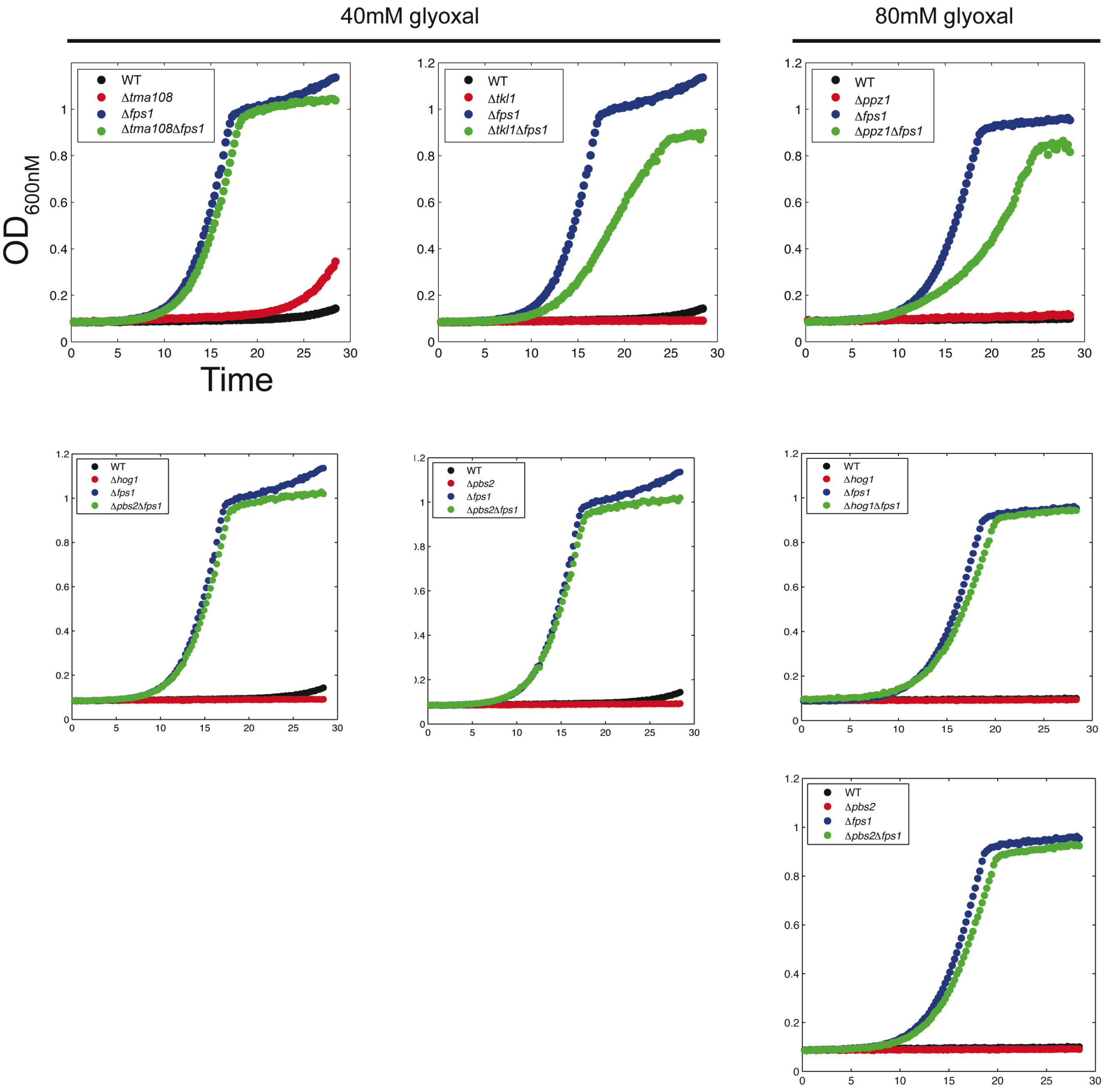

Figure S2 Loss of *Δfps1* suppresses glyoxal sensitivity of *Δtma108*, *Δtkl1*, *Δppz1*, *Δpbs2*, *Δhog1* mutants at higher glyoxal concentrations.
