# Supplementary material for: A Global Perspective of the Genetic Basis for Carbonyl Stress Resistance
Source: G3 (Bethesda). 2011 Aug 1;1(3):219–31. doi: 10.1534/g3.111.000505 (PMC3276133; doi:10.1534/g3.111.000505)
Supplement: Supporting Information [file supp_1.3.219_TableS5.pdf]

Table S5 Plasmids used in this study

Plasmids	Characteristics	Source
P5476	High copy number *LEU2* marker	Charlie Boone
P5476-*ZDS2*	High copy number *LEU2* marker, *ZDS2*-ORF plus regulatory regions	Charlie Boone
P5476-*GLO2*	High copy number *LEU2* marker *GLO2*-ORF plus regulatory regions	Charlie Boone
P5476-*THR1*	High copy number *LEU2* marker *THR1*-ORF plus regulatory regions	Charlie Boone
P5476-*YPR1*	High copy number *LEU2* marker *YPR1*-ORF plus regulatory regions	Charlie Boone
P5476-*SLG1*	High copy number *LEU2* marker *SLG1*-ORF plus regulatory regions	Charlie Boone
P5476-*PDE2*	High copy number *LEU2* marker *PDE2*-ORF plus regulatory regions	Charlie Boone
P5476-*GRE3*	High copy number *LEU2* marker *GRE3*-ORF plus regulatory regions	Charlie Boone
P5476-*PBS2*	High copy number *LEU2* marker *PBS2*-ORF plus regulatory regions	Charlie Boone
P5476-*RHO2*	High copy number *LEU2* marker *RHO2*-ORF plus regulatory regions	Charlie Boone
P5476-*MRPL28*	High copy number *LEU2* marker *MRPL28*-ORF plus regulatory regions	Charlie Boone
P5476-*STP1*	High copy number *LEU2* marker *STP1*-ORF plus regulatory regions	Charlie Boone
P5476-*HLR1*	High copy number *LEU2* marker *HLR1*-ORF plus regulatory regions	Charlie Boone
P5476-*MIG3*	High copy number *LEU2* marker *MIG3*-ORF plus regulatory regions	Charlie Boone
P5476-*MIG2*	High copy number *LEU2* marker *MIG2*-ORF plus regulatory regions	Charlie Boone
P5476-*ADH6*	High copy number *LEU2* marker *ADH6*-ORF plus regulatory regions	Charlie Boone
P5476-*YGR126W*	High copy number *LEU2* marker *YGR126W*-ORF plus regulatory regions	Charlie Boone
P5476-*SSK1*	High copy number *LEU2* marker *SSK1*-ORF plus regulatory regions	Charlie Boone
P5476-*GIS3*	High copy number *LEU2* marker *GIS3*-ORF plus regulatory regions	Charlie Boone
P5476-*HOG1*	High copy number *LEU2* marker *HOG1*-ORF plus regulatory regions	Charlie Boone
P5476-*SSK2*	High copy number *LEU2* marker *SSK2*-ORF plus regulatory regions	Charlie Boone
P5476-*ERG5*	High copy number *LEU2* marker *ERG5*-ORF plus regulatory regions	Charlie Boone
P5476-*GIS2*	High copy number *LEU2* marker *GIS2*-ORF plus regulatory regions	Charlie Boone
P5476-*MED8*	High copy number *LEU2* marker *MED8*-ORF plus regulatory regions	Charlie Boone
P5476-*PBN1*	High copy number *LEU2* marker *PBN1*-ORF plus regulatory regions	Charlie Boone
pRS426-*GLO1*	High copy number *URA3* marker *GLO1*-ORF plus regulatory regions	This study
pUG23-*FPS1*GFP	Low copy number *HIS3* marker *FPS1*-ORF driven by Met25 promoter	This study
